# Passive maternal exposure to environmental microbes selectively modulates the innate defences of chicken egg white by increasing some of its antibacterial activities

**DOI:** 10.1186/1471-2180-13-128

**Published:** 2013-06-07

**Authors:** Larbi Bedrani, Emmanuelle Helloin, Nicolas Guyot, Sophie Réhault-Godbert, Yves Nys

**Affiliations:** 1 UR83 Recherches Avicoles, Institut National de la Recherche Agronomique, Nouzilly, France; 2 UMR1282, Infectiologie et Santé Publique, Institut National de la Recherche Agronomique, Nouzilly, France

**Keywords:** Chicken, Egg, Germ free, Innate immunity, Antimicrobial protein

## Abstract

**Background:**

Egg defence against bacterial contamination relies on immunoglobulins (IgY) concentrated in the yolk and antimicrobial peptides/proteins predominantly localized in the egg white (EW). Hens contaminated with pathogenic microorganisms export specific IgYs to the egg (adaptative immunity). No evidence of such regulation has been reported for the antimicrobial peptides/proteins (innate immunity) which are preventively secreted by the hen oviduct and are active against a large range of microbes. We investigated whether the egg innate defences can be stimulated by the environmental microbial contamination by comparing the antimicrobial activity of EW of hens raised in three extreme breeding conditions: Germ-free (GF), Specific Pathogen Free (SPF) and Conventional (C) hens.

**Results:**

The difference in the immunological status of GF, SPF and C hens was confirmed by the high stimulation of IL-1β, IL-8 and TLR4 genes in the intestine of C and SPF groups. EW from C and SPF groups demonstrated higher inhibitory effect against *Staphylococcus aureus* (13 to 18%) and against *Streptococcus uberis* (31 to 35%) as compared to GF but showed similar activity against *Salmonella* Enteritidis, *Salmonella* Gallinarum, *Escherichia coli* and *Listeria monocytogenes*. To further investigate these results, we explored putative changes amongst the three main mechanisms of egg antimicrobial defence: the sequestration of bacterial nutrients, the inactivation of exogenous proteases and the direct lytic action on microorganisms. Lysozyme activity, chymotrypsin-, trypsin- and papain-inhibiting potential of EW and the expression of numerous antimicrobial genes were not stimulated suggesting that these are not responsible for the change in anti-*S. aureus* and anti-*S. uberis* activity. Moreover, whereas the expression levels of IL-1β, IL-8 and TLR4 genes were modified by the breeding conditions in the intestine of C and SPF groups they were not modified in the magnum where egg white is formed.

**Conclusions:**

Altogether, these data revealed that the degree of environmental microbial exposure of the hen moderately stimulated the egg innate defence, by reinforcing some specific antimicrobial activities to protect the embryo and to insure hygienic quality of table eggs.

## Background

Eggs contain a large variety of nutrients and are a source of balanced proteins with high nutritional value for humans. They are widely consumed throughout the world and are used in food processing for their technological properties. Their hygienic quality is of major concern especially when used as a raw nutrient. An egg is sterile when laid in non-pathological conditions but after being laid, it can be contaminated despite its efficient protective barriers [1,2]. The egg is protected physically by the eggshell and chemically by antibodies, known as IgYs, mainly concentrated in the egg yolk [3] and throughout the egg by numerous peptides and proteins possessing antimicrobial properties [4]. These molecules constitute an innate immunity and are secreted “preventively” by the hen ovary into the egg yolk to protect the embryo, and by the other oviduct segments into the other egg compartments (egg white, eggshell membranes and eggshell). Egg antimicrobial proteins and peptides operate via three main mechanisms: (i) sequestration of essential nutrients from bacteria by the chelation of minerals (iron) or from vitamins (biotin) by proteins such as ovotransferrin and avidin, respectively [5]; (ii) inactivation of exogenous proteases necessary for microbial metabolism and invasion of host tissues (egg antiproteases including cystatin, ovomucoid and ovoinhibitor) [6]; (iii) direct lytic action on microorganisms by lysozyme or peptides belonging to the defensin family whose actions lead to the disruption of the bacterial cell wall [7]. The innate immunity of eggs is modulated by several parameters. Among these, genetic control has been demonstrated as the anti-*Staphylocccus aureus* and the *anti-Salmonella* Enteritidis activity of egg white have heritabilities (values reflecting the extent to which a phenotype is influenced by the genotype) of 0.16 and 0.13 respectively [8]. Hen physiology, in particular hen age [9] or immune-stimulatory treatments [10] have been reported to alter activities of some effectors of the egg innate chemical defence including lysozyme and anti-proteases. To our knowledge, there is no evidence demonstrating that antimicrobial peptide or protein concentrations and/or their activities might be modified by the exposure of the hen to pathogenic and/or non-pathogenic environmental microbes, as demonstrated for yolk antibodies [3,11]. This question is of interest since EU-directive 1999/74 became effective at the beginning of 2012. Conventional cage housing has been banned and only eggs issuing from alternative breeding systems are marketable. This major change in the hen breeding system has modified the hen microbial environment [12,13] and might increase egg shell contamination, as suggested by some comparisons between cage and non-cage breeding systems [14,15]. Therefore, we explored whether the microbial environment of the hen influences innate immunity by increasing the oviduct secretion of antimicrobial proteins into the egg white, and its antibacterial activity. Any modification in egg antimicrobial molecules which are much less selective for specific pathogens compared to IgY and are potentially active against a wide range of microbes including bacteria, viruses or parasites [4] might positively impact on the hygienic quality of table eggs.

With this objective in mind, we studied three experimental models reflecting large differences in hen microbial environment and immunological status: Germ-free animals (GF), Specific Pathogen Free animals (SPF), and Conventional hens (C). Germ-free (GF) animals are reared in sterile conditions and show a wide range of defects in the development of their immune system and in antibody production, particularly intestine IgA. In GF mice, the normal immune function is also impaired at the tissue, cellular and molecular levels in the absence of gut microbiota [16,17]. SPF females are not subjected to any vaccination treatment and are bred in strictly controlled environments that are free of pathogens. In contrast, the conventional hens are vaccinated against highly virulent microorganisms and are reared in commercial facilities where environmental microbes are diverse and might even include pathogens.

In the present study, we have used these extreme breeding conditions to explore the impact of the hen microbial environment on the modulation of innate immunity in the egg, as reflected by egg white antibacterial activity.

## Results

### Maintaining germ-free, specific pathogen free and conventional hens

GF hens were bred in two isolators and strict conditions were applied to keep them in a sterile environment. The absence of bacteria in the isolators was checked twice a month throughout the experimental period using the referenced method (PFIE-NT-0061) on fresh faeces directly sampled from the cloaca and inoculated into two cultivation media: thioglycolate resazurine broth and heart infusion broth. Our strategy was partially successful as our analysis did not reveal the initial presence of any bacteria; however, a contamination by *Penicillium* was detected when the hens were 18 weeks of age.

Specific pathogen free hens (SPF) were kept in strict hygienic conditions and were certified free of pathogens as determined by the control procedure of the experimental infectiology platform (PFIE-FE-0172). Our conventional hens were issued from the same line and flock than SPF hens but were reared with commercial laying hens at 16 weeks for 10 weeks before egg sampling. However, they have not been vaccinated against virulent microorganisms as carried out for commercial birds.

### Gene expression in jejunum and caecum by RT-qPCR

**Figure 1:**
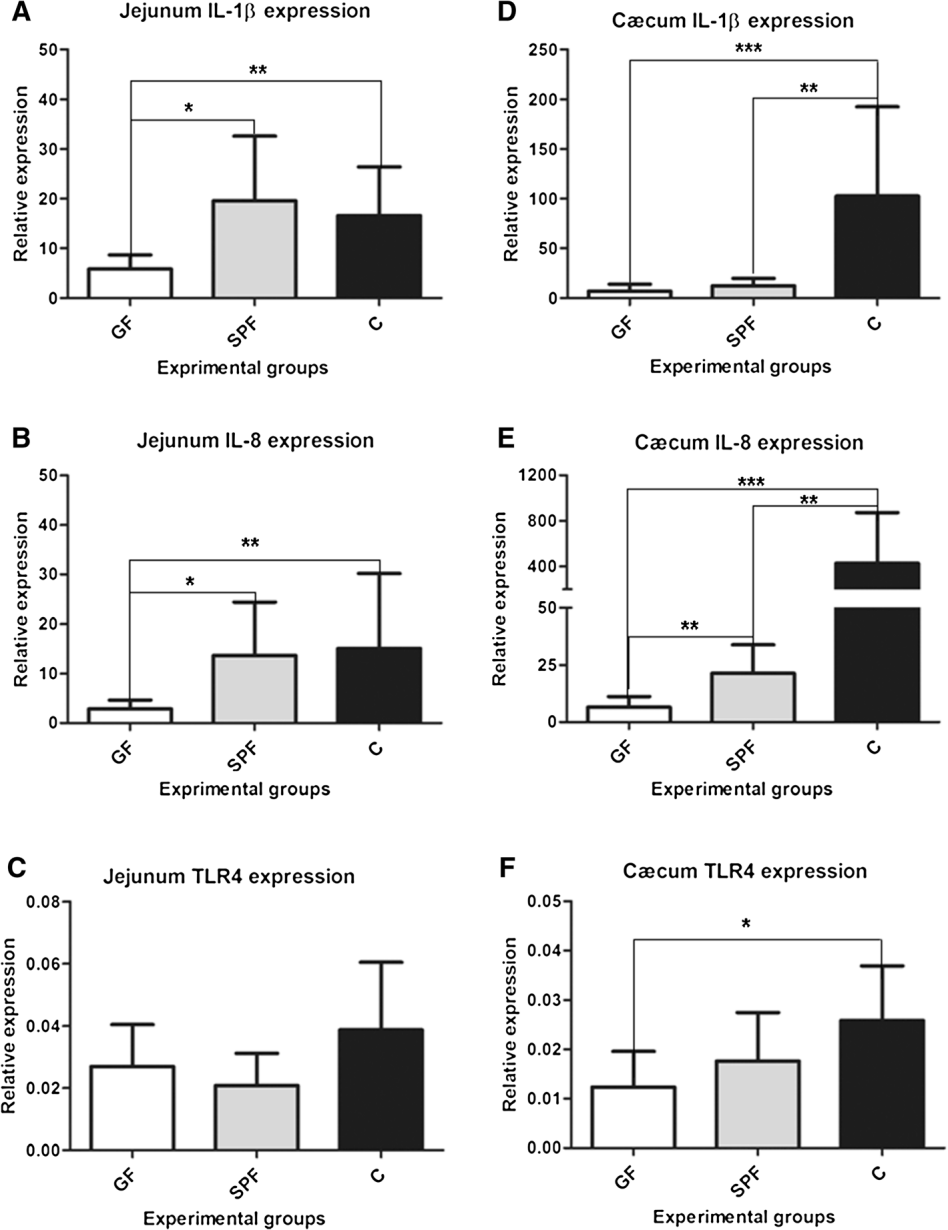
**Gene expression levels in the jejunum and the caecum of GF, SPF and C groups.** In the jejunum, the gene expression levels of IL-1β and IL-8 (**A** and **B** respectively) were higher in C and SPF as compared to GF. In the cæcum, IL-1β and IL-8 were overexpressed in C group as compared to SPF and GF. IL-8 and TLR4 mRNA level were also higher respectively in SPF and C groups compared to GF. (n = 8; mean ± standard deviation;*p < 0.05; **p < 0.01; ***p < 0.001). IL-1β and IL-8 data (**A**, **B**, **D**, **E**) were analysed using the Kruskal-Wallis test followed by the Mann–Whitney test; TLR4 data (**C**, **F**) were analysed using one-way ANOVA followed by the Bonferroni-Dunn test.

To better appreciate the immunological status of the three experimental groups, we first investigated the expression of interleukin-1 beta (IL-1β), interleukin-8 (IL-8) and Toll-like receptor-4 (TLR4) genes in the jejunum and the cæcum, as presented in Figure 1. In the jejunum, there was a 1.8- and 2.3-fold increase in IL-1β gene expression (Figure 1A), in C (p < 0.005) and SPF groups (p < 0.05), compared to GF. Similarly, the IL-8 gene (Figure 1B) expression was 3.7 and 4.2 times higher in C and SPF groups as compared to GF group (p < 0.05 and p < 0.005, respectively). However, no statistically significant difference was observed between C and SPF for both IL-1β and IL-8 in the jejunum. The TLR4 expression levels remained similar amongst the three experimental groups.

In the cæcum (Figures 1D, E, F), IL-1β was overexpressed in the C group by more than 6- and 13-fold as compared to SPF (p < 0.01) and GF (p < 0.005), respectively. The mRNA levels between these latter groups were similar. The IL-8 gene expression was also higher in the C group as compared to both SPF and GF groups. IL-8 expression was higher in C hens by more than 19-fold (versus SPF, p < 0.005) and 64-fold (compared to GF, p < 0.001). The SPF group demonstrated higher IL-8 mRNA levels (elevated more than 3-fold) compared with GF (p < 0.01). Finally, the TLR4 gene expression was higher in conventional hens (C) (1.6 fold; p < 0.05) as compared to GF hens, but not different from SPF hens.

### Egg white antibacterial activity

**Figure 2:**
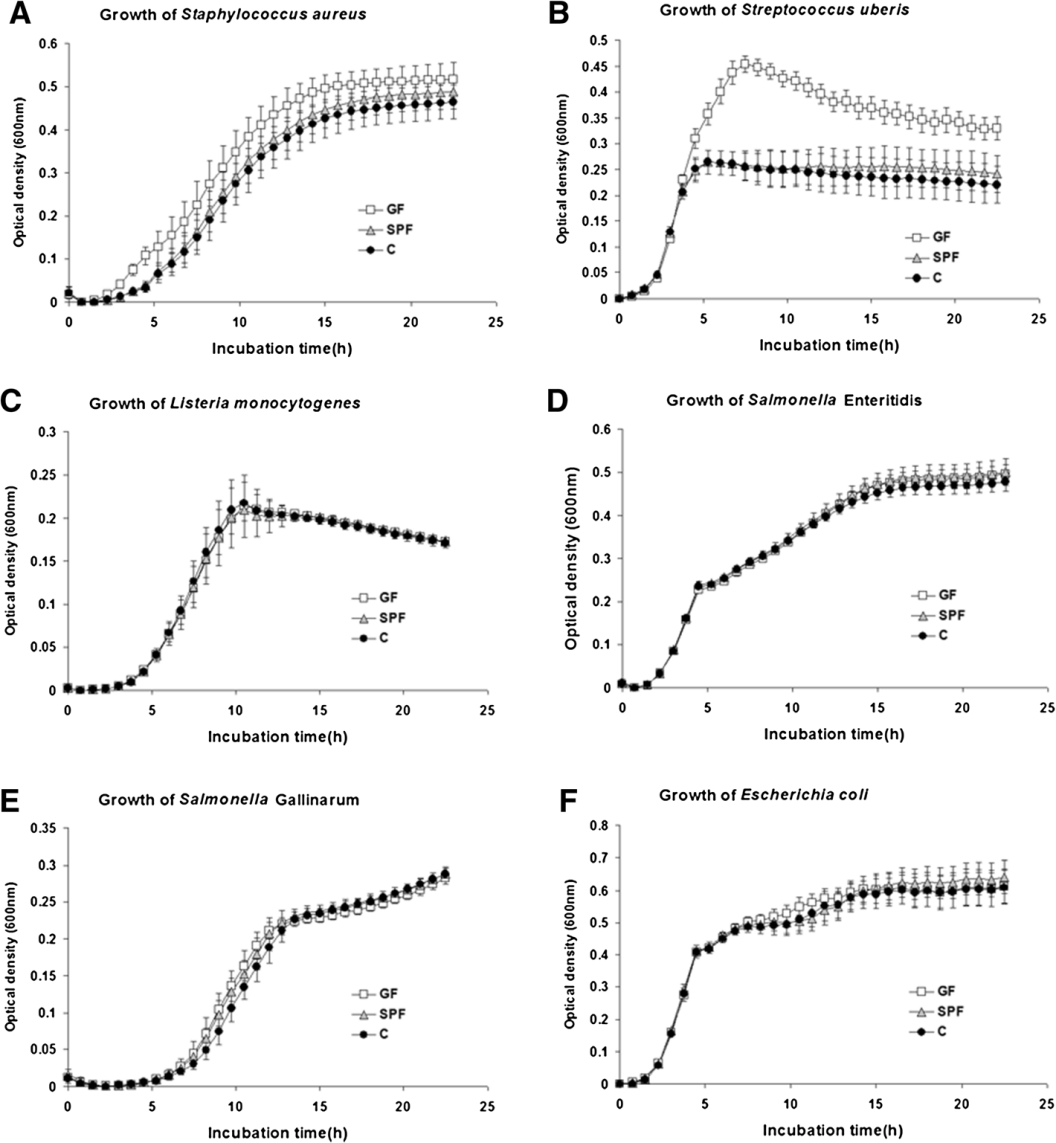
**Growth of several bacterial strains in presence of GF, SPF and GF egg whites.** The growth inhibition of *S. aureus* (**A**), *S. uberis* (**B**) was significantly higher in C and SPF hens as compared to GF (p < 0.001) while no differences were recorded among these three groups regarding the growth of *L. monocytogenes* (**C**), *S.* Enteritidis (**D**), *S.* Gallinarum (**E**) and *E. coli* (**F**). Germ free (GF), Specific Pathogen Free (SPF) and conventional (C) hens (n = 10, mean ± standard deviation).

The growth curves obtained after cultivating *S. aureus*, *S. uberis*, *L. monocytogenes*, *S.* Enteritidis, *S.* Gallinarum and *E. coli* in the presence of diluted egg white from C, SPF and GF groups are shown in Figure 2. The mean values of the total growth (area under curve) are reported in Table 1. The growth of *S. aureus* (Figure 2A) was significantly lower by 17.6% (p < 0.001) and 13.0% (p < 0.05) respectively for the egg whites derived from C and SPF groups, as compared to the GF hens. Similarly, the growth of *S. uberis* (Figure 2B) was lower by 34.8% in the C group (p < 0.001) and by 31.4% (p < 0.01) in SPF as compared with GF hens. No difference was observed between C and SPF hens when measuring the growth of *S. aureus* and *S. uberis*. On the other hand the growth of *L. monocytogenes* (Figure 2C), *S.* Enteritidis (Figure 2d), *S.* Gallinarum (Figure 2E) and *E. coli* (Figure 2F) in presence of egg white were similar for the three experimental treatments (Table 1).

**Table 1:** Growth of six bacterial species in egg white of GF, SPF and conventional hens

**Bacterial species**	**Germ free hens**	**Specific pathogen free hens**	**Conventional hens**	**P value**
Staphylococcus aureus**	7.4 ± 0.7 a*	6.4 ± 0.7 b	6.1 ± 0.5 b	<0.001
***Streptococcus uberis***	7.3 ± 0.3 a	5.0 ± 0.6 b	4.7 ± 0.8 b	<0.001
***Listeria monocytogenes***	3.1 ± 0.1	3.0 ± 0.2	3.0 ± 0.1	0.91
***Salmonella *Enteritidis**	7.5 ± 0.2	7.7 ± 0.3	7.4 ± 0.2	0.11
***Salmonella *Gallinarum**	3.2 ± 0.2	3.3 ± 0.2	3.1 ± 0.1	0.18
***Escherichia coli***	10.6 ± 0.6	10.6 ± 0.6	10.4 ± 0.3	0.48

*mean **areas under the growth curves** ± standard deviation, n = 10 Means with different letters are different (p < 0.05). Data were analysed using one-way ANOVA followed by the Bonferroni-Dunn test.

**Staphylococcus aureus D8 618.29, *Streptococcus uberis* 3029C MC, *Listeria monocytogenes* strain EGDe, *Salmonella* Gallinarum 229 K and *Salmonella enterica.* Enteritidis ATCC 13076 were provide d by INRA (Nouzilly) and Avian *Escherichia coli* CIRMBP-0096 was provided by the International Center of Microbial Resources dedicated to Pathogenic Bacteria (Nouzilly).

### Protein concentration and pH

Protein concentration and pH mean values for C, SPF and GF groups are shown in Table 2.

**Table 2:** Protein concentration, pH, lysozyme and protease inhibiting activities of egg whites (GF, SPF and C hens)

**Measurements**	**Germ-free hens**	**Specific pathogen free hens**	**Conventional hens**	**P value**
**Protein concentration (mg/ml)**	111 ± 14	119 ± 14	116 ± 6	0.24
**pH**	8.41 ± 0.10 a*	8.54 ± 0.11 b	8.60 ± 0.15 b	<0.001
**Lysozyme activity (U/mg)**	110200 ± 51220	96700 ± 29820	101700 ± 35120	0.74
**Remaining protease inhibition activity (% of control)**				
**Anti-papain activity**	45.4 ± 5.3	43.7 ± 5.5	41.8 ± 3.5	0.26
**Anti-trypsin activity**	46.4 ± 2.9	46.3 ± 4.6	45.9 ± 2.9	0.95
**Anti-chymotrypsin activity**	44.2 ± 4.6	48.6 ± 5.2	48.8 ± 4.9	0.07

*Values are mean ± standard deviation, n = 10. Means with different letters are different (p < 0.05). The pH values were analysed using the Kruskal-Wallis test followed by the Mann–Whitney test; all other data were analysed using one-way ANOVA followed by the Bonferroni-Dunn test.

The pH of GF hen albumen was lower compared to those from C and SPF hens; the differences are 0.19 unit higher in C compared with GF groups (p < 0.001), and 0.13 higher in SPF egg white compared with GF eggs (p < 0.001). The mean albumen pH values were similar between C and SPF egg whites.

Total protein quantification of egg whites did not reveal any statistically significant difference between GF, C and SPF groups (P > 0.5).

### Egg white lysozyme and protease inhibition activities

Lysozyme is a muramidase responsible for the cleavage of the bond between the N-acetyl-muramic acid and N-acetyl-glucosamine. These two molecules are found in the peptidoglycan of bacterial cell wall. Under our experimental conditions, lysozyme activities of the egg whites were similar for GF, SPF and C groups, as shown in Table 2.

Anti-proteases can impair bacterial invasion by inhibiting bacterial proteases which are major virulence factors. Anti-papain and anti-trypsin activities showed no differences between the three experimental groups of hens (Table 2). We detected, however, a trend for a higher anti-chymotrypsin activity in C and SPF groups as compared to GF groups (+10.3% and +10.0% for C and SPF, as compared to the GF group, respectively, which was not significant; p = 0.07).

### Gene expression in the reproductive tract

**Figure 3:**
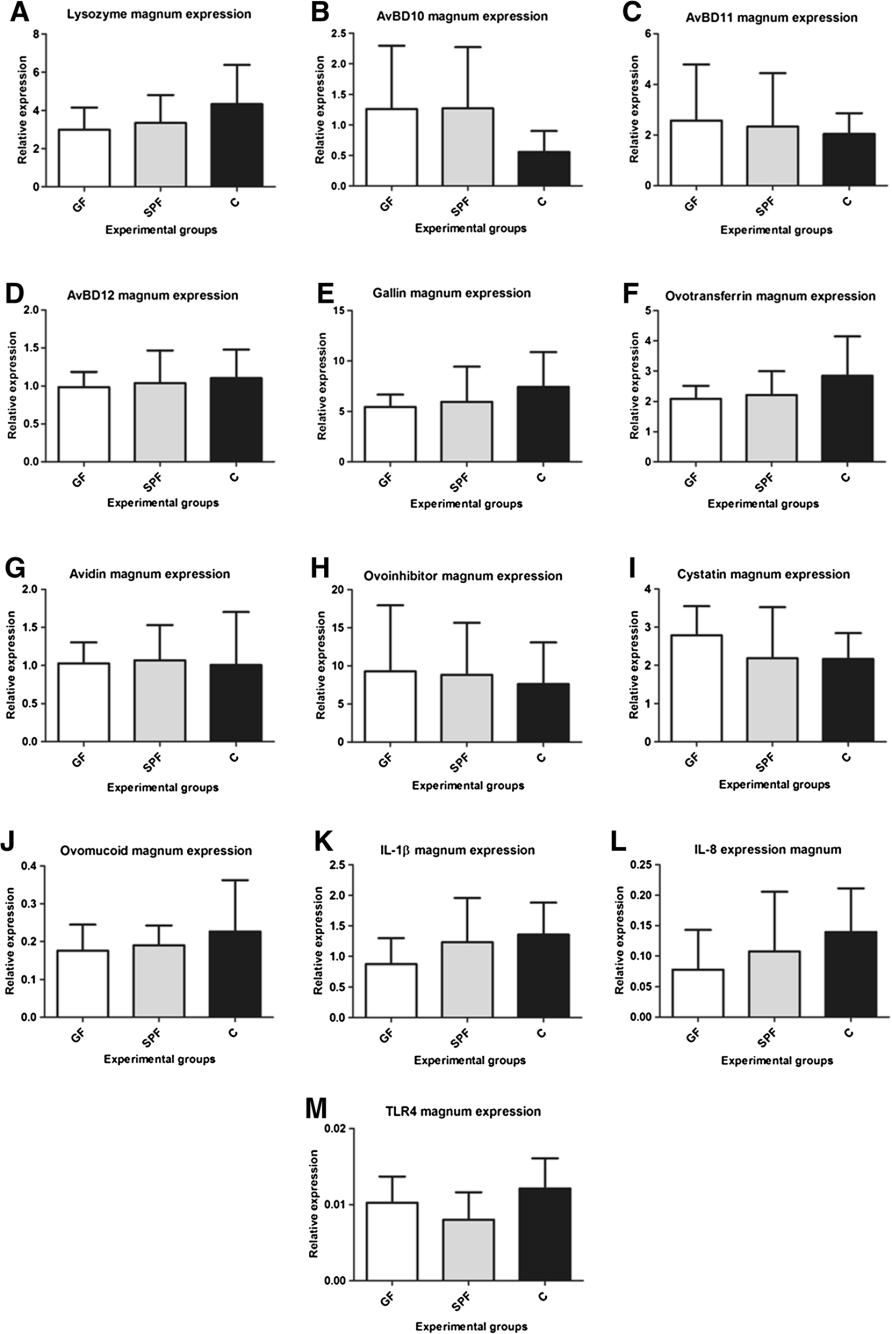
**Genes expression levels in the magnum of GF, SPF and C groups.** Gene expression levels of lysozyme (**A**), AvBD 10 (**B**), AvBD 11 (**C**), AvBD 12 (**D**), gallin (**E**), ovotransferrin (**F**), avidin (**G**), ovoinhibitor (**H**), cystatin (**I**), ovomucoid (**J**), IL1-β (**K**), IL8 (**L**) and TLR4 (**M**) in the magnum as assessed by RT-qPCR showed no difference among the three experimental groups GF, SPF and C (n = 8; mean ± standard deviation, * p < 0.05). Data in A, D, G, H, I, K, L and M were analysed using one-way ANOVA followed by the Bonferroni-Dunn test; data in B, C, E, F and J were analysed using the Kruskal-Wallis test followed by the Mann–Whitney test.

We analysed in the three experimental groups the expression of genes encoding proteins whose function is to prevent bacterial growth either by direct lytic action, or by chelating nutrients or by inhibiting bacterial proteases (Table 3). We also analysed the expression of genes encoding some cytokines and TLR4 (the lipopolysaccharide receptor) to gain insight into some regulators of the immune response in the oviduct. Figure 3 shows the expression levels of lysozyme (A), avian beta defensin (AvBD) 10 (B), AvBD11 (C), AvBD12 (D), gallin (E), ovotransferrin (F), avidin (G), ovoinhibitor (H), cystatin (I), ovomucoid (J), IL-1β (K), IL-8 (L) and TLR4 (M) in the magnum tissue of the GF, SPF and C groups. The magnum is the part of the oviduct which synthesizes and secretes egg white proteins. The expression of the genes coding for the proteins having direct lytic action on bacteria, lysozyme (A), AvBD10 (B), AvBD11 (C), AvBD12 (D) and gallin (E) was similar in the magnum of the three experimental groups. Ovotransferrin (F), avidin (G) are respectively iron and biotin chelators present in the egg white. Their mRNA expression in the magnum of GF, SPF and C groups did not differ significantly. Similarly, the gene expression of ovoinhibitor (H), cystatin (I) and ovomucoid (J) which are antiproteases was similar among the three groups. Finally and in the same way, the expression of genes coding for IL-1β, IL-8 and TLR4 showed no difference between the three experimental groups.

**Table 3:** Functions, genes accession numbers and primers used for magnum and egg white proteins transcription studies

**Protein function**	**Genes**	**Primers**	**Accession number**
**Proteins with direct lytic action on bacteria**	**Lysozyme**	F-GGGAAACTGGGTGTGTGTTGCA	[GenBank:bFJ542564.1]
		R-TCTTCTTCGCGCAGTTCACGCT	
	**AvBD 10**	F-GCTCAGCAGACCCACTTTTC	[GenBank:NM_001001609.1]
		R-GTTGCTGGTACAAGGGCAAT	
	**AvBD 11**	F-ACTGCATCCGTTCCAAAGTC	[GenBank:NM_001001779.1]
		R-TGTGGCTTTCTGCAATTCTG	
	**AvBD 12**	F-GGGGATTGTGCCGAGTGGGG	[GenBank:NM_001001607.2]
		R-TGCTGGAGGTGCTGCTGCTC	
	**Gallin**	F-CTCCAGCCTCGCTCACAC	[GenBank:FN550409.1]
		R-TTGAGAGGAGGGGATGACAC	
**Chelating proteins**	**Ovotransferrin**	F-GACTTGCAGGGCAAGAACTC	[GenBank:NM_205304.1]
		R-GCTGGCAGAGAAAAACTTGG	
	**Avidin**	F-CTGCATGGGACACAAAACAC	[GenBank:NM_205320.1]
		R-TTAACACTTGACCGCAGCAG	
**Protease inhibiting proteins**	**Cystatin**	F-ACAACTTGCCCCAAGTCATC	[GenBank:NM_205500.2]
		R-GGCAGCGATACAATCCATCT	
	**Ovoinhibitor**	F-TAAGGATGGCAGGACTTTGG	[GenBank:NM_001030612.1]
		R-GAGTTTGCCACCAGTGGTTT	
	**Ovomucoid**	F-TGCAGTCGTGGAAAGCAACGG	[GenBank: FJ227543.1]
		R-GCTGAGCTCCCCAGAGTGCGA	
**Cytokines**	**Interleukin 1**	F-AGTGGCACTGGGCATCAAGG	[GenBank:HQ329098.1]
		R-TGTCGATGTCCCGCATGACG	
	**Interleukin 8**	F-CTGCGGTGCCAGTGCATTAG	[GenBank:HM179639.1]
		R-CCATCCTTTAGAGTAGCTAT	
	**TLR4**	F- TTCAAGGTGCCACATCCAT	[GenBank:AY064697]
		R- TAGGTCAGACAGAGAGGATA	
	**TBP**	F-GCGTTTTGCTGCTGTTATTATGAG	[GenBank:NM_205103.1]
		R-TCCTTGCTGCCAGTCTGGAC	

## Discussion

The primary protection of the egg after being laid relies firstly on a physical defence (the eggshell and the eggshell membranes) and secondly on chemical defences mainly present in the egg white, but also in other compartments. IgY, IgM and IgA [11] participate with numerous major proteins [18] and newly identified minor proteins and peptides [4] in the innate defences of the egg. While IgY concentration have been shown to vary in egg yolk depending of the nature and degree of antigen exposure of hen [19], no evidence in the literature explored whether the antimicrobial peptides/proteins of the egg are modulated by the microbial environment of the hen. The present study demonstrates the regulation of some egg white antimicrobial activities by microbial environment of the hen.

This investigation used an experimental design based on the comparison of three extreme conditions of rearing laying hens: germ-free (GF), specific pathogen-free (SPF) and conventional (C) conditions. GF hens are characterized by the absence of microbiota at the intestinal level. This influences their metabolism and intestinal morphological parameters [20]. SPF hens are raised in strictly hygienic conditions and are not vaccinated. Due to the absence of any interactions with other pathogenic microorganisms, the SPF model is frequently used to explore immunological responses to pathogenic or vaccine antigens [21,22]. In contrast, C laying hens are bred under commercial conditions and might occasionally be exposed to pathogens. These contrasting breeding conditions provide extremely wide qualitative and quantitative variations in terms of bacterial populations in contact with the hens: the absence or presence of surrounding microbes and gut microbiota, for the GF or C groups respectively, and an intermediate group, the SPF hens, hosting a controlled microbiota in a pathogen-free environment. The maintenance of GF hens until they are sexually mature (4–5 months) and beyond requires efficient isolators, sterilized food and water, and qualified animal handlers. These constraints could partly explain why such an animal model has never been used before. In our attempt, the non-contamination of GF hens was not successfully achieved. An agent, *Penicilium*, was detected at month four, in two independent isolators, but more importantly, in spite of this fungal contamination, the hens remained free of bacteria relevant to our initial objective.

The GF group definitively showed different immunological statuses compared to the C and SPF groups, as reflected by higher expressions of IL-1β, IL-8 and TLR4 genes in the jejunum and cæcum of these groups, compared to the GF group. IL-1β and IL-8 are two pro-inflammatory cytokines which are often used as markers of inflammation [23]. TLR4 is a host cell membrane receptor that detects lipopolysaccharide from Gram-negative bacteria and elicits innate immune response following bacterial infection. The difference in expression levels of IL-1β, IL-8 among the three groups was larger in the cæcum (2- to 64-fold) than in the jejunum (2- to 4-fold) in the SPF and C groups as compared to the GF group. Such expected differences are probably due to the bacterial load, which is much higher in the cæcum than in the jejunum [24]. In contrast, no differences in IL-1β, IL-8 and TLR4 gene expression were observed in the oviduct (magnum) between the experimental groups. Under normal non-pathogenic conditions, the magnum and the other segments of the hen oviduct (infundibulum, isthmus and uterus) constitute an aseptic environment in which the egg is formed in a 24 hour period [2]. These aseptic conditions are probably responsible for the absence of pro-inflammatory gene induction. Altogether, these observations suggest that the presence or absence of microflora and associated stimuli, at the intestinal or oviduct levels respectively, directly influences the local inflammatory state and the tissue expression of IL-1β, IL-8 and TLR4 genes.

The egg white is the largest compartment of the egg in terms of variety and concentration of antimicrobial proteins. Among the major egg white antimicrobial proteins are ovotransferrin and lysozyme, which are active against Gram-negative and Gram-positive bacteria [4,25]. Apart from these major egg white compounds, a number of minor molecules with potent antimicrobial activities have recently been identified and further characterized. Of these, we characterized the antibacterial activities of two peptides of the beta-defensin family, namely gallin and the avian beta-defensin [26,27]. While gallin is active against *E. coli*, AvBD11 possesses a broad spectrum of antibacterial activities against both Gram-positive and Gram-negative bacteria, The ability of the hen to modulate these compounds in response to microbial environments has not been explored. Egg whites of the C and SPF groups had greater inhibitory activities on the growth of *S*. *aureus* and *S*. *uberis* (Figure 2A, B, P < 0.01) than those of the GF hens. In contrast, anti-*Salmonella* (*S.* Enteritidis and *S.* Gallinarum), anti-*E. coli* and anti-*L. monocytogenes* activities were similar in the egg whites of all three experimental groups. Our results demonstrated that the breeding conditions of hens have an impact on some of the antibacterial properties of their eggs, according to the degree of bacterial contamination of their environment. However, the response seemed specific to certain bacterial strains, suggesting that it might result from change in some antimicrobial egg molecules with a particular spectrum of activity, predominantly toward Gram-positive bacteria in our study. In order to give some insight into the putative mechanisms at the origin of the increased egg white antibacterial activity against *S. aureus* and *S. uberis* observed in SPF and C groups, we further analysed the level and/or activity of a panel of proteins representative of the main modes of action of egg antimicrobials (chelating, antiprotease and lytic effects). That was carried out by quantifying egg white activities or magnum gene expression of proteins representative of this diversity of antibacterial actions.

The main bacteriolytic molecule of the egg white is the lysozyme. This well-studied cationic protein is an enzyme catalysing the cleavage of peptidoglycan, a major compound of Gram positive bacterial cell walls. No variation between GF, SPF and C was observed for the lysozyme-mediated lytic activity of egg whites. This is in agreement with the lysozyme amounts, which appeared to be constant within all experimental groups, as assessed by western blot (data not shown) and with previous findings showing the genetic stability of lysozyme concentration in the egg white [8]. In the opposite, hen age and acute administration of different immunostimulatory substances to hens modulate its activity [9,10]. However, our results were coherent with unmodified anti-*L. monocytogenes* activity. Egg white exerts a potent bactericidal activity against *L. monocytogenes* and the main egg component possessing anti-*Listeria* activities is the lysozyme. In contrast, *L. monocytogenes*, *S. aureus* and *S. uberis* seemed to be less sensitive to the egg white antimicrobial activities and grew in less diluted egg white. A number of *S. aureus* strains are known to develop resistance to lysozyme, whereas the activity of egg white lysozyme on *S. uberis* strains requires further study. The fact that no variation between GF, SPF and C was observed for the lysozyme-mediated lytic activity of egg whites supports the hypothesis that enhanced anti-*S. aureus* and anti-*S. uberis* activities in SPF and C egg white are not related to lysozyme, but most probably to additional compound(s). Egg white contains numerous bactericidal molecules including the avian defensins. These cationic peptides can disrupt the bacterial membrane, resulting in the cell lysis [7,28]. Thus, gallin and avian beta-defensins (AvBDs) 10, 11 and 12 which have been detected in the egg white by proteomic analysis [29] and/or in the magnum at transcriptional level [30] are alternative candidates to explain a change in antimicrobial activities. The quantification of these peptides was not possible because neither specific antibodies nor quantitative ELISA kits are available. Variation at the transcriptional level was therefore analysed by RT-qPCR in the magnum as a potential marker for relative protein synthesis between experimental groups. Previous studies showed that hens intravenously injected with lipopolysaccharide showed a transitory increased expression of AvBD10, AvBD11 and AvBD12 in the vagina [30,31]. In our steady-state experimental conditions, even if C and SPF hens were more challenged immunologically than GF hens, their magnum showed no stimulation of AvBD10, AvBD11, AvBD12 and gallin expression, suggesting that these molecules are not responsible for the increased antimicrobial activity observed in the egg white. Therefore, the higher anti-*S. aureus* and anti-*S. uberis* activities in the egg white of C hens did not appear to rely on AvBD10, AvBD11, AvBD12 and gallin.

Egg white contains large amounts of chelating molecules with antimicrobial activities, the most representative being ovotransferrin and avidin. Ovotransferrin was quantified both at the protein (western blot, data not shown) and transcriptional levels, while avidin was assessed only at the transcriptional level. No modifications in any of the three hen groups were revealed for these molecules. It is believed that the most efficient antimicrobial molecule against Gram-negative bacteria *E. coli* and *S.* Enteritidis in the egg is ovotransferrin via its iron depriving mechanism [25,32]. *S.* Enteritidis is of major concern in public health as it is considered as the first foodborne disease agent in eggs and egg products [2]. This bacterium is capable of invading the intact egg when laid and, via different mechanisms, of withstanding the antibacterial molecules as well as the harsh pH conditions in the egg white during its storage [33]. The absence of variation in *S*. Enteritidis growth in any of the three conditions was consistent with our observations showing that ovotransferrin was not modified, either at protein or transcriptional levels.

Egg white antiproteases might play a role in egg innate immunity by exhibiting antimicrobial activities. Cystatin is a potent antimicrobial, active against a variety of bacteria including *Escherichia coli* and *S. aureus*[34]. Two other egg antiproteases, ovomucoid and ovoinhibitor, are known to inhibit bacterial peptidases [35,36] in spite of limited data regarding their antimicrobial properties. In particular, their effect on *S. aureus* is yet unknown. Likewise, there is no data in the literature demonstrating anti-*S. uberis* properties for ovomucoid, ovoinhibitor and cystatin. In our study, the analysis of egg white antiprotease activities and magnum gene expression of these molecules was of interest as staphylococci and streptococci are bacteria known to secrete extracellular peptidases that presumably play some role in virulence. In particular, *S. aureus* produces and releases to the extracellular milieu several enzymes belonging to distinct classes of proteases, such as serine- (Protease V8 or SspA), cysteine- (Staphopains A and B, also known as ScpA and SspB) and metallo- (Aureolysin Aur) proteases [37]. *S. uberis* produces extracellular proteases that are involved in the regulation of biofilm formation [38]. Our results showed that global anti-trypsin, anti-chymotrypsin and anti-papain-like protease activities were not influenced by the microbial environment of hens. Moreover, gene expression analyses of ovoinhibitor, cystatin and ovomucoid in the magnum did not show any differences among the three experimental groups. These observations suggest that increased egg white activities against *S. aureus* and *S. uberis* do not rely on these egg antiproteases.

The egg white pH affects global egg white antimicrobial activity. High pH values are bactericidal for *S. aureus*[39] and are correlated with anti-*S.* Enteritidis activity [40]. Egg white pH was slightly higher in C (+0.19) and SPF (+0.13) groups as compared to GF (pH = 8.41). However, for this magnitude of changes, there was no correlation between pH and anti-*S. aureus* or anti-*S. uberis* activities (correlation coefficients were respectively −0.16 and −0.50; p > 0.1) so this parameter is unlikely to explain the bacterial growth inhibition.

Our observation that only two out of the six bacteria studied responded to the treatment, suggests that the effect results from some specific egg molecules. However, our attempt to identify the molecular origin of the change in the egg white antimicrobial activities observed for *S. aureus* and *S. uberis* was not fruitful. It strongly suggests that additional egg components, not investigated in the present study, are involved in this regulation. The sequencing of the hen’s genome and the development of proteomic [29,41,42] and transcriptomic [43] approaches reveal hundreds of minor peptides and proteins expressing a large range of biological functions including protection against diverse pathogens (bacteria, viruses, fungi) [4] in the different egg compartments. An alternative explanation for the difficulty in identifying the minor egg molecules responsible for the increased antibacterial effect towards *S. aureus* and *S. uberis* is that we explored the gene expression of candidate proteins, and not the egg protein or peptide levels or activities in the eggs. However, by using such extreme experimental situations (GF, SPF, C), this strategy should be valid and this was confirmed by the dramatic changes observed for interleukins at the intestinal level. It is obvious, however, that numerous alternative candidates amongst the newly identified molecules may be at the origin of the observed changes, including histone-like proteins or lipolysaccharide-binding proteins [4].

## Conclusions

The present study shows evidence that the microbial environment of the hen modulates some of the antibacterial activities of the egg white, independently of the pH. The change in the antibacterial activity remains however of moderate magnitude and concerns only a limited number of bacteria (2 out of 6). In particular, the microbial contamination of the hen environment changed anti-*S. aureus* and anti-*S. uberis* egg white activities, whereas anti-*S.* Enteritidis egg white activity was not affected. The restricted bacterial spectra affected by the bacterial environment suggested a change in some of the minor egg protein or peptides for which it would be useful to develop quantitative methods for measuring their level and antibacterial activity. The absence of anti-*Salmonella* modulation by the hen in response to microbial milieu underlines the importance of keeping the environment free of *Salmonella* to reduce egg contamination risks in the alternative breeding systems emerging in Europe.

## Methods

### Experimental design

#### Ethics statement

All experiments, including all animal-handling protocols, were carried out in accordance with the European Communities Council Directives of 24 November 1986 (86/609/EEC) concerning the practice for the care and Use of Animals for Scientific purposes and the French ministerial decree 87848 of 19 October 1987 (revised on 31 May 2001) on Animal experimentation under the supervision of authorized scientists (authorization # 6563, delivered by the DDPP, direction départementale de la protection des populations, d’Indre et Loire). The experimental infectiology platform (PFIE, B37-176-3, INRA, Nouzilly; France) where the birds were kept has the agreement for rearing birds and for the euthanasia of experimental animals (decree N° SA0900850 of 25^th^ August 2009, delivered by the Préfecture d’Indre et Loire following the inspection of the Department Direction of Veterinary Services (DDPP, direction départmentale de la protection des populations). The sampled eggs were non embryonic (table egg).

### Animals

Thirty eight PA12 Leghorn hens were bred in the PFIE. They were divided into three experimental groups: A) a Germ-Free (GF) group (n = 8) where chicks were hatched and raised in a sterile environment (two pressurised isolators) until sexual maturity and initiation of egg production. The hens were fed X-ray sterilized diet (SDS Dietex, Argenteuil, France) and sterilized water for the entire duration of the trail (more than 6 months). B) a Specific Pathogen Free (SPF) group (n = 12) corresponding to hens housed in individual cages in a pressured chamber and bred/maintained in strictly hygienic conditions to prevent any contact with known pathogenic microorganisms. C) a Conventional (C) group (n = 18) that was kept under conventional breeding conditions but in individual cages. C hens were initially PA12 SPF females which were transferred at 16 weeks of age to conventional breeding facilities hosting commercial laying ISA-Brown hens in their production period. C hens however remained unvaccinated until the end of the trial.

The lightening program consisted of 16 hours of light and 8 hours of obscurity. Food and water were provided *ad libitum.*

### Albumen processing

A total of 80 eggs were collected per experimental group of hens (20 to 30 weeks of age). Eggs were checked visually to remove cracked eggs and then stored at 4°C for 48 hours before sampling. After this period, the eggs were flamed using absolute ethanol and broken under sterile conditions. The albumens were separated from yolks, homogenized using an ultraturax device (T 18 basic ULTRA-TURRAX®, IKA-Werke, Staufen, Germany) aliquoted into microtubes and stored at −20°C until use. Ten pools of eight egg whites were constituted per treatment and used to carry out the antibacterial assays and other analysis.

### Antimicrobial activity assay

A turbidimetric approach was used to study the antimicrobial activity of the egg whites against several pathogenic bacterial strains. The automated turbidometer Bioscreen C Reader (Bioscreen C ®, Thermo Fisher Scientific, Saint-Herblain, France) has been used in various studies to evaluate the impact of antibacterial molecules on growth parameters of bacteria and has shown a good accordance with estimates obtained by plate count [44,45]. *Staphylococcus aureus D8 618.29* and *Streptococcus uberis* 3029C MC were kindly provided by Pascal Rainard (INRA, UMR1282, Nouzilly, France). *Listeria monocytogenes* strain EGDe, *Salmonella* Gallinarum 229 K and *Salmonella enterica* Enteritidis ATCC 13076 were kindly provided by Philippe Velge (INRA, UMR1282, Nouzilly, France). Avian *Escherichia coli* CIRMBP-0096 was provided by the International Center of Microbial Resources dedicated to Pathogenic Bacteria (CIRM-BP, INRA Tours, France). *Listeria monocytogenes* and *Streptococcus uberis* were grown in tryptic soy broth and brain heart infusion, respectively. All the remaining bacteria were cultured in Mueller-Hinton broth. The bacterial strains (frozen in 25% glycerol) were cultured overnight at 37°C prior to the bacterial assay. The following day, an aliquot of the overnight culture was then inoculated in fresh broth and cultured at 37°C with agitation (320 rpm) until reaching the optical density (OD) corresponding to mid-exponential growth phase previously defined according to whole growth curves determination studies (data not shown). An aliquot of 50 μL of diluted albumen sample (in 50 mM Tris–HCl, pH 7.4) were deposited in triplicate in sterile 100-well honeycomb microplates and mixed with 50 μL of a bacterial suspension (2×10^6^ CFU/mL in 2X broth) obtained by diluting the mid-exponential growth phase culture. The final bacterial concentration was 10^6^ CFU/ml per well. Final egg white dilutions were 1/120 for *L. monocytogenes*, 3/16 for *S. uberis* and 3/8 for the remaining strains. Culture media and egg-white samples used in the study were verified for the absence of bacterial contamination. The plates were then incubated at 37°C for 22.5 hours in an automated OD recorder (Bioscreen C®, Thermo Fisher Scientific, Saint-Herblain, France).

The OD values were measured for each well at 600 nm every 45 min after 10 seconds of high speed shaking, and means were calculated from the three replicates. The quantification of antimicrobial activities for each albumen sample was based on the calculation of area under the growth curves as determined by the following formula: area  =  t * ((OD_1_/2 + OD_final_/2) + sum(OD_2_;  OD3 … OD_final - 1_)), where t is the time interval between two measurements, OD_1_ the first measured OD and OD_final_ the last measured OD. We considered the area under the growth curves to facilitate the comparison of the impact of egg whites on bacterial growth between the different groups tested (GF, SPF and C). To guaranty that this value really reflects the growth parameters, we choose to limit its calculation in the OD interval where the reliability of the relationship between OD and the numbers of CFU/ml has been highlighted by preliminary studies.

### pH measurement and protein quantification

The pH of the albumen was measured using a laboratory pH meter (pH meter BASICS 20+, Crison, France) after homogenisation of the egg white pools. Total protein concentration was quantified using the Coo Protein Assay Reagent (Interchim, Montluçon, France) on 5 μL of a 1/200 dilution of egg white, according to the manufacturer’s recommendation.

### Antiprotease activities of egg white

The protease-inhibition activities of egg white were assessed against trypsin, chymotrypsin and papain. The microassays were based on the use of chromogenic peptidic substrates covalently coupled at their carboxy-terminal extremity to para-nitroanilide (pNA). T-Glu-Phe-Arg-pNA, Succinyl-Ala-Ala-Pro-Phe-pNA and pGlu-Phe-Leu-pNA (Sigma Aldrich, Saint-Quentin Fallavier, France) were used to study the trypsin, chymotrypsin and papain inhibitory activities of the egg white, respectively. The assays were performed in 96-well plates in 200 μL final volume per well, with 50 mM Tris–HCl 50 mM NaCl; pH 7.4 as a buffer for both trypsin and chymotrypsin assays. The papain assays utilized 0.1 M Bis Tris, 1 mM EDTA, 2 mM 1,4-dithio-DL-threitol, pH 6. Twenty μL of 1/64000, 1/200 and 1/20 egg white dilutions were incubated 1 h at 30°C with 130 μL of trypsin, chymotrypsin and papain, respectively. Then 50 μL of the appropriate peptidic substrate (2 mM) were added. Final enzyme concentrations were 0.8 nM for both trypsin and chymotrypsin and 0.4 μM for papain. The quantities of egg white used in each protease assay were chosen in order to obtain 50% to 60% inhibition as compared to a control containing only the substrate and the enzyme. The hydrolysis of each substrate was recorded during 30 min by continuous monitoring of the absorbance of pNA at 410 nm.

### Lysozyme activity assay

Lysozyme activity of the egg whites was determined using the lysoplate method [46] modified for 96-well plates [5]. Briefly, lyophilised *Micrococcus lysodeikticus* (Sigma Aldrich, Saint-Quentin Fallavier, France) was suspended in PBS (0.5 mg/ml) and kept at a temperature of 45–50°C. Fifteen μL of the albumen dilution (1/200 in 50 mM Tris–HCl, pH 7.5) was mixed with 150 μL of the bacterial suspension in each well of a 96 well plate maintained on ice. The absorbance at 420 nm of each sample was measured at 25°C over 6 minutes using a microplate reader (Infinite®, Tecan, Lyon, France). Lysozyme activity of each albumen sample was determined by recording the absorbance decrease in *Micrococcus lysodeikticus* culture. The log absorbance values recorded within 3 min for each egg white sample showed linear curves whose slopes were reported to each egg white protein concentration in the assay. The results are expressed as Unit/mg of egg white protein where one Unit corresponds to a decrease of OD by 0.01 per minute at 450 nm.

### Tissues sampling and gene expression analysis

#### Tissue sampling

Tissue sampling was performed on eight hens of each experimental group. A lethal intravenous injection of pentobarbital sodium (CEVA santé animale, France) was used for the sacrifice of the animals (Authorization # 7323). Samples (n = 8) of the mucosal layers of magnum, jejunum and cæcum were collected in cryotubes, snap frozen and stored at −80°C until use.

### Gene expression analysis

Total mRNA from tissues was extracted using RNA Now (Biogentec, Seabrook, TX) according to the manufacturer’s recommendations. RNA concentrations were determined by measuring the absorbance at 260 nm using a spectrophotometer (Nanodrop® ND1000, Labtech, Paris, France). RNA integrity was verified by electrophoresis in 1% agarose gel using ethidium bromide. Reverse-transcription was performed using RNase H-MMLV reverse transcriptase (Superscript II, Invitrogen, Cergy Pontoise, France) and random hexamers (Amersham, Orsay, France). The resulting cDNA was amplified by real-time RT-PCR (RT-qPCR) using SYBR Green I (ROCHE SAS, Boulogne-Billancourt, France). Primers of the genes are listed in Table 3.

### Statistical analysis

Data are presented as mean ± standard deviation. Statistical analyses were carried out using Statview version 5.0 (SAS Institute, Cary, North Carolina). The homogeneity of the variances were checked using Barttlet test for equal variances. When the latter was no significant (p > 0.05), data were analysed using one way ANOVA followed by Bonferroni-Dunn test for the pair-wise comparison. When the variances were different (Barttlet test, p < 0.05) data were analysed using the Kruskal-Wallis test followed by a Mann Whitney test for the pair-wise comparisons.

## Competing interests

The authors declare that they have no competing interests.

## Authors’ contributions

LB, EH contributed to the strategy, the experimental design, and planning of the study. LB carried out the experiments and analyses, interpreted data and wrote the first draft of the paper. EH, NG, SR contributed to the interpretation of data and to the writing of the paper. YN conceived the research program focused on regulation of egg innate immunity. He was involved in the strategy, the experimental design, data interpretation and was fully involved in the writing of the paper. All authors have read and approved the final manuscript.
